# The Effect of Insecticide Treated Nets (ITNs) on Plasmodium falciparum Infection in Rural and Semi-Urban Communities in the South West Region of Cameroon

**DOI:** 10.1371/journal.pone.0116300

**Published:** 2015-02-25

**Authors:** Tobias O. Apinjoh, Judith K. Anchang-Kimbi, Regina N. Mugri, Delphine A. Tangoh, Robert V. Nyingchu, Hanesh F. Chi, Rolland B. Tata, Charles Njumkeng, Clarisse Njua-Yafi, Eric A. Achidi

**Affiliations:** 1 Department of Biochemistry and Molecular Biology, University of Buea, Buea, Cameroon; 2 Department of Zoology and Animal Physiology, University of Buea, Buea, Cameroon; 3 Department of Microbiology and Parasitology, University of Buea, Buea, Cameroon; 4 Department of Medical Laboratory Science, University of Buea, Buea, Cameroon; 5 Department of Animal Biology and Physiology, University of Yaounde I, Yaounde, Cameroon; Institute of Tropical Medicine, JAPAN

## Abstract

Insecticide Treated Nets (ITNs) have been shown to reduce morbidity and mortality, but coverage and proper utilization continues to be moderate in many parts of sub-Saharan Africa. The gains made through a nationwide free distribution were explored as well as the effect on malaria prevalence in semi-urban and rural communities in south western Cameroon. A cross sectional survey was conducted between August and December 2013. Information on net possession, status and use were collected using a structured questionnaire while malaria parasitaemia was determined on Giemsa-stained blood smears by light microscopy. ITN ownership increased from 41.9% to 68.1% following the free distribution campaign, with 58.3% (466/799) reportedly sleeping under the net. ITN ownership was lower in rural settings (adjusted OR = 1.93, 95%CI = 1.36–2.74, p<0.001) and at lower altitude (adjusted OR = 1.79, 95%CI = 1.22–2.62, p = 0.003) compared to semi-urban settings and intermediate altitude respectively. Conversely, ITN usage was higher in semi-urban settings (p = 0.002) and at intermediate altitude (p = 0.002) compared with rural localities and low altitude. Malaria parasitaemia prevalence was higher in rural (adjusted OR = 1.63, 95%CI = 1.07–2.49) compared to semi-urban settings and in those below 15 years compared to those 15 years and above. Overall, participants who did not sleep under ITN were more susceptible to malaria parasitaemia (adjusted OR = 1.70, 95%CI = 1.14–2.54, p = 0.009). Despite the free distribution campaign, ITN ownership and usage, though improved, is still low. As children who reside in rural settings have greater disease burden (parasitemia) than children in semi-urban settings, the potential gains on both reducing inequities in ITN possession as well as disease burden might be substantial if equitable distribution strategies are adopted.

## Introduction

Malaria due to *Plasmodium falciparum* remains one of the most important causes of morbidity and early mortality in endemic regions of sub-Saharan Africa [1]. Current malaria control strategies involve early diagnoses and treatment of infected individuals and the reduction of human-mosquito contact rates through vector control efforts [2]. Malaria-related mortality, morbidity and economic loss could, therefore, be averted if the available effective preventive and treatment interventions are made universally accessible to those in need [3]. Nevertheless, inadequate access to information, healthcare and antimalarial resources results in the inability to properly implement malaria interventions [4]. Furthermore, disparities in access between rural and urban locations exist [4], with rural areas found to have less access to malaria control interventions [5, 6].

Insecticide treated nets (ITNs) and indoor residual spraying (IRS) have both been demonstrated to reduce malaria [7–10] and, to date, are the mainstay for controlling malaria vectors and associated malaria transmission [1,10]. Nevertheless, long lasting insecticide-treated bed nets (LLINs)/ITNs are the major and most promising components of the selective vector control strategies [11,12]. In fact, a massive scale-up in malaria control programmes between 2008 and 2010 resulted in the provision of ITNs to protect more than 578 million people at risk and the concomitant reduction in mortality from 985,000 in 2000 to 781,000 in 2009 [13]. Therefore, the government of Cameroon embarked on a scaling-up of ITN coverage in 2011, in line with the Roll Back Malaria (RBM) recommendation of universal coverage [14,15].

However, bed nets as a tool for malaria control can present challenges, such as coverage, proper use and replacement of old and torn nets [16]. Recent data [17] suggest that net possession and use remain low in Cameroon, with only 36% ITN ownership and 21% of children below 5 years reportedly sleeping under an ITN. The coverage and proper utilization of this malaria preventive measure in the country may be limited by the lack of sustainable distribution and issues related to replacement of nets, seasonality of malaria, and poor knowledge of the community about the link between mosquitoes and malaria [18]. The possible shift in local malaria epidemiology also necessitates the evaluation of their proper use and effectiveness in ensuring their long-term benefit [19–21]. In Addition, establishing determinants of infection and evaluating the effectiveness of vector control interventions can identify possible ways to improve malaria control [22]. The World Health Organization [23], therefore, recommends periodic surveys to assess whether populations at risk receive sufficient LLINs/ITNs and that these are properly used. Nevertheless, assessments on possession and utilization of the LLINs in Cameroon have been limited [24] and have not been done in the study area since the last free distribution from district health offices to the community.

While challenges to increasing ITN ownership may diminish as a result of the expansion of large-scale distribution efforts, ITN impact on transmission will be minimized if they are not properly and consistently used, especially among populations vulnerable to increased malaria morbidity and mortality, such as children and pregnant women [25]. In addition, considerable disparity has been observed between ITN possession and use [26–28].

Although ITNs have been shown to reduce morbidity and mortality in numerous controlled trials [8], the protective effect of the tool on malaria parasitaemia warrants further investigation. ITNs have been shown to reduce asexual parasitaemia prevalence in children under 5 years old [29] as well as increase protection for community members not sleeping under any bednet at all and decrease malaria prevalence in surrounding areas following community-wide use [30]. Nevertheless, no association was observed between *P*. *falciparum* infection and reported individual use of an ITN the previous night in Benin [31] and Tanzania [32]. Furthermore, ITNs have recently been shown to be limited in reducing the number of malaria vectors entering the houses even at high coverage levels [33].

This study uses observational data from large cross sectional surveys conducted in 2013 to assess net possession and investigate the protective effect of ITNs on malaria infection in semi-urban and rural communities in the South West Region of Cameroon.

## Materials and Methods

### Ethics Statement

The study was approved by the Institutional Review Board of the Faculty of Health Sciences, University of Buea, Cameroon (N^0^: 2013-03-0153) while administrative authorization was obtained from the South West Regional Delegation of Public Health. Written informed consent or assent was obtained from all participants or the parents/guardians for those below 21 years of age.

### Study Area

**Fig 1:**
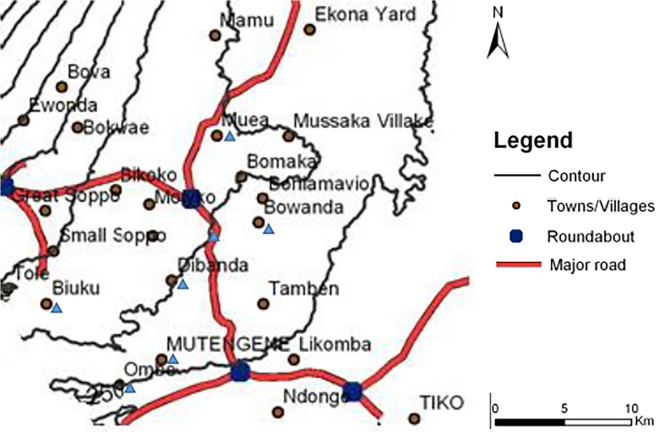
Map of the study area. Localities on the slope of Mt. Cameroon included in the survey are indicated by blue triangles.

The study was conducted in seven localities on the eastern slope of Mt Cameroon, with varying malaria transmission profiles and geographic features (Table 1). All selected sites were geo-located using a handheld GPS (eTrex, Vista, Garmin, USA); two communities below 200 m were considered to be at low altitude while five between 385–636 m were at intermediate altitude (Fig. 1). The terrain rises from the Atlantic ocean at the Gulf of Guinea, gradually increasing from Ombe through Mutengene to 800–1,200 meters in Buea. The area has many streams that empty into the ocean, including, Ombe and Onge that flow southeast and northwest respectively [34]. The forested equatorial climate, modified by the ocean and mountain, comprises two seasons: a short dry season (November–March) and a long rainy season (March-November). This is characterized by fairly constant temperatures that vary from 18⁰C in August to 35⁰C in March [34,35]. The relative humidity (75–80%), average annual rainfall (2625 mm) and precipitation (2,000–10,000 mm) are relatively high [35].

**Table 1:** Geographical and parasitologic characteristics of the study sites in the Mount Cameroon area.

Locality	Site	Geographic coordinates	Altitude		n	ITN (%)		Malaria		
			masl^&^	class		Ownership	Use	PP* (%)	Morbidity (%)	GMPD^$^
Rural	Tole	4°11′N, 9°24′E	636	Intermediate	129	41.9	36.4	28.7	9.3	2021
	Ombe Native	4°06′N, 9°29′E	135	Low	75	79.7	71.6	20.0	2.7	1820
Semi—Urban	Muea	4°17′N, 9°30′E	533	Intermediate	131	76.3	73.7	13.0	3.8	1357
	Mile 16—Bolifamba	4°13′N, 9°30′E	485		99	75.8	61.6	14.1	1.0	997
	Mile 15—Buea	4°11′N, 9°30′E	397		138	91.3	87.7	21.0	7.4	1790
	Mile 14—Dibanda	4°11′N, 9°30′E	385		119	56.3	43.7	21.0	1.7	948
	Mutengene	4°08′N, 9°30′E	197	Low	109	67.0	33.0	22.0	3.7	3750

*PP = parasite prevalence; ^$^GMPD = geometric mean parasite density per microliter of blood; ^&^masl = metres above sea level

Malaria transmission is intense and perennial in the area, with parasitaemia higher in the rainy seasons [35] and at lower altitude [36]. *P*. *falciparum* accounts for most of the infections while *Anopheles gambiae* is the dominant, most aggressive and most active of the three malaria vectors (*A*. *gambiae*, *A*. *funestus* and *A*. *nili*) [34,35]. Infection rates by *A*. *gambiae* are as high as 287 infective bites/person/year, with overall Entomological Inoculation Rates (EIR) of 3.93 infective bites/person/night [34].

Although the indigenes of this area are of the Bakweri tribe and part of the Bantu ethnic group [37], its fertile volcanic soils and vast plantations have attracted people from other regions of the country, mainly from the Semi-Bantu ethnic group of the north west. Plank houses predominate in the villages while cement block houses are more common in the semi-urban settings. Subsistence farming is the mainstay of the village communities, which rely mainly on agriculture for their livelihood.

### National ITN Distribution Campaign

The government of the Republic of Cameroon undertook a nationwide free LLITN distribution campaign from health facilities to all households in the country at the end of 2011. The objective of the campaign was to provide an ITN, with a lifespan of five years, to all household beds or an LLITN for every two individuals per household, to a maximum of three ITNs per household.

### Study Population and Sample Survey

This is a cross-sectional community—based study that was conducted between August and December 2013. Communities were first identified as rural and semi-urban and then randomly selected based on differences in altitude. Authorization to conduct the survey was then sought from Chiefs or Quarter heads and a community was only investigated with their approval. The population was then adequately sensitized on the project objectives, methods and possible benefits/risks through elites and/or community leaders. Participants were then invited to a central enrollment point and only enrolled if they or their caregivers/guardians gave written informed consent/assent. All individuals that had been living in the community during the free ITN distribution campaign were eligible for enrollment. Nevertheless, only resident adults were interviewed in either English/French (national languages) or pidgin (local language) to document ethnodemography and household ITN ownership and usage.

### Data acquisition and Definition of terms

A structured questionnaire was used to record ownership, utilisation, status (either torn or good), source and approximate date of acquisition of the bed net as well as gender, age and area of residence of all participants. The participant’s axillary temperature was then measured using a digital thermometer, with current fever defined as body temperature ≥ 37.5°C taken during the survey. A sample of blood (2 ml) was then collected from each participant, by venepuncture, into an EDTA tube (BD Vacutainer Systems, Plymouth, UK) for parasitological analysis. An ITN was defined as any long-lasting insecticidal net, any bednet factory-treated with insecticide and obtained less than 36 months ago, or any bednet treated with insecticide less than 36 months ago. ITN use were defined as reportedly sleeping under a bednet or ITN the previous night. Coverage was defined as the proportion of individuals reportedly having an ITN over their sleeping space. Ethnicity was defined as the self-reported ethnic group of the mother (or father if the mother’s could not be obtained). Parasitemia was defined as presence of asexual *P*. *falciparum* parasites on a thick blood film whereas malaria was defined as current fever together with parasitaemia.

### Malaria parasitaemia determination

Thick and thin blood smears were prepared following standard procedures and stained with 10% Giemsa (Sigma, St. Louis, USA). The malaria parasitaemia status and density were determined under oil immersion with the 100x objective, 10x eyepiece of a binocular Olympus microscope (Olympus Optical Co., Ltd, Japan) while the *Plasmodium* species was identified on the thin blood smear. A smear was only considered negative if no malaria parasites were seen in 50 high power fields. With each positive smear, the level of parasitaemia was estimated by counting the parasites against at least 200 leucocytes and assuming a leucocyte count of 8000 per microlitre to calculate the number of parasites/μl blood [36].

### Statistical Analyses

All data were entered into Excel and analysed using SPSS Statistics 20 for windows (SPSS Inc, Chicago USA). Levels of parasitaemia were log-transformed before analysis. The significance of difference in prevalence were explored using the Pearson’s χ^2^ test whereas the differences in group means were assessed using Student’s t—test or analyses of variance (ANOVA). Association analysis of malaria prevalence and ITN usage was undertaken by logistic regression, using as covariates altitude, location, gender and age group. A difference giving a P value ≤ 0.05 was considered statistically significant.

## Results

**Fig 2:**
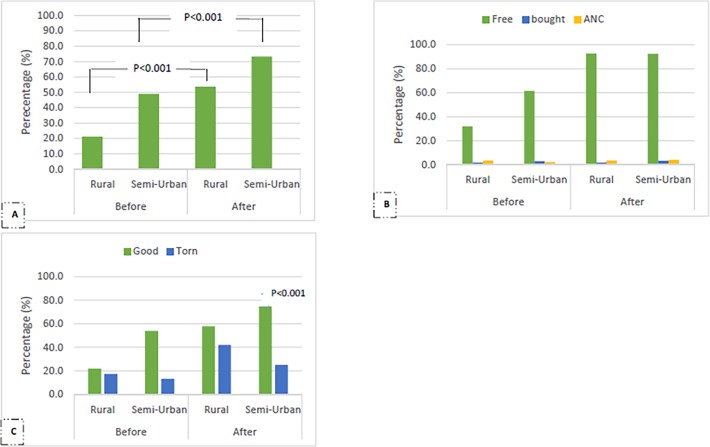
Reported ownership, quality and source of ITN of participants (n = 800) who started using bednets before and after the free distribution campaign.

The geographical and parasitological characteristics of the study sites are shown in Table 1. A total of 800 individuals were enrolled mainly from semi-urban settings (74.5%), semi-bantu ethnic group (78.4%), intermediate altitude (77.1%) and above 15 years old (44.9%) (Table 2). The mean age (± SD) of the participants was 19.48 ± 18.50 years (range: 4 months—90 years). The reported ownership, quality and source of ITN of study participants who started using nets before and after the free distribution campaign is shown on Fig. 2. Three hundred and thirty five (41.9%) and 545 (68.1%) of the study participants reportedly owned bednets before and after the campaign respectively. This increment in ITN ownership was observed significantly in both (p<0.001) rural (33.2%, 66/199) and semi-urban (24.4%, 144/591) settings (Fig. 2). Most of the bednets before and after the campaign [(91.3%, 304/333) vs (92.8%, 504/543)] were obtained for free from the public sector. A significant proportion of the ITNs obtained before the campaign (77.3%, 259/335) were reportedly in good condition at the time of the study compared to 71.3% (388/544) of nets obtained after the campaign. However, the proportion of good nets (74.7%, 325/435) was higher (p<0.001) than torn nets (25.3%, 110/435) in the semi-urban settings following the campaign (Fig. 2). Nevertheless, the ratio of good to torn nets was similar in rural settings.

**Table 2:** Relationship between sociodemographic characteristics of study participants and ITN ownership and usage in the South West Region of Cameroon.

Variable	Subclass	% (n)	ITN Ownership					ITN Usage				
			% (n)	Unadjusted P value	OR	95% CI	Adjusted P value	% (n)	Unadjusted P value	OR	95% CI	Adjusted P value
**Age groups (years)**	< 5	22.1 (177)	68.4 (121)	0.623	0.92	0.61–1.38	0.675	57.1 (101)	0.084	1.18	0.80–1.73	0.412
	5–9	20.4 (163)	66.3 (108)		1.21	0.79–1.85	0.384	58.9 (96)		1.04	0.69–1.56	0.863
	10–14	12.4 (99)	73.7 (73)		1.22	0.70–2.16	0.483	69.7 (69)		1.12	0.65–1.94	0.688
	≥ 15	44.9 (359)	69.9 (251)		Ref			55.4 (199)		Ref		
**Gender**	Male	43.8 (350)	70.0 (245)	0.720	1.06	0.76–1.47	0.739	60.0 (210)	0.396	0.95	0.70–1.30	0.753
	Female	56.3 (450)	68.8 (309)		Ref			57.0 (256)		Ref		
**Ethnicity**	Semi—Bantu	78.4 (537)	65.2 (350)	0.327	0.74	0.50–1.09	0.127	54.2 (291)	**0.008**	1.68	1.16–2.44	**0.007**
	Bantu	21.6 (148)	60.8 (90)		Ref			41.9 (62)		Ref		
**Locality**	Rural	25.5 (204)	55.7 (113)	**< 0.001**	1.93	1.36–2.74	**< 0.001**	49.3 (100)	**0.002**	0.83	0.59–1.16	0.272
	Semi—Urban	74.5 (596)	74.0 (441)		Ref			61.4 (366)		Ref		
**Altitude**	Low	22.9 (183)	48.6 (89)	0.350	1.79	1.22–2.62	**0.003**	48.6 (89)	**0.002**	1.17	0.83–1.66	0.366
	Intermediate	77.1 (616)	72.1 (132)		Ref			61.2 (377)		Ref		

OR = Odds Ratio; CI = Confidence Interval; Ref = Reference group; Boldface numbers indicate significant p values

### ITN Ownership and Usage

**Fig 3:**
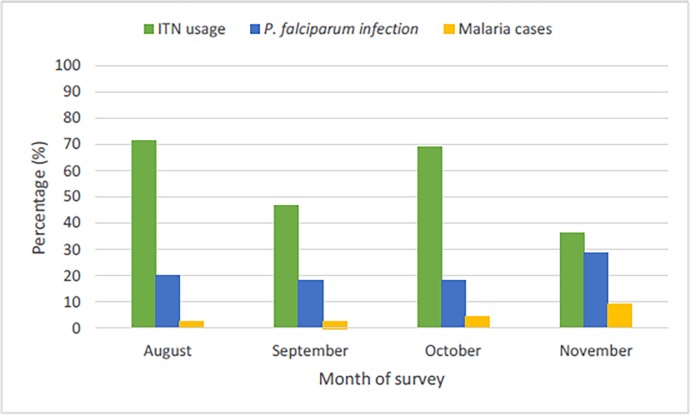
Temporal distribution of ITN use, *P*. *falciparum* infection and malaria cases among participants from south western Cameroon.

The overall reported ITN ownership and usage was 69.3% (554/799) and 58.3% (466/799) respectively. ITN ownership and usage was independent of age and gender (Table 2). ITN ownership was lower in rural settings (adjusted OR = 1.93, 95%CI = 1.36–2.74, p<0.001) and at lower altitudes (adjusted OR = 1.79, 95%CI = 1.22–2.62, p = 0.003) compared to semi-urban settings and intermediate altitude respectively. However, ITN usage was significantly (p<0.001) associated with month of survey (Fig. 3) and was higher in semi-urban settings (p = 0.002) and at intermediate altitude (p = 0.002) compared with rural localities and low altitude in univariate analysis. Nevertheless, the higher usage of ITNs by members of the semi-bantu ethnic group remained (adjusted OR = 1.68, 95%CI = 1.16–2.44, p = 0.007) after adjusting for age, altitude and locality.

### Malaria Parasitaemia

The overall prevalence of parasitaemia was 20.1% (161/800), with a geometric mean parasite load of 1721 parasites/μl blood (range: 80–193,655). 4.5% (36/798) of the study participants actually had malaria in the community. Additionally, parasitaemia prevalence was higher (p<0.001) in participants below 15 years (29.6%, 130/439) compared to those above 15 years (8.6%, 31/360). The parasitaemia density was independent of gender, ethnicity and locality but varied with age group and altitude (Table 3). The prevalence of parasitaemia did not vary significantly with altitude or ethnicity or month of survey but was higher (p = 0.040) in males compared to females and in rural (adjusted OR = 1.63, 95%CI = 1.07–2.49) compared to semi-urban settings. Furthermore, participants below 14 years old were more susceptible to malaria parasitaemia compared to those 15 years and above (Table 3) following multivariate analysis. The number of malaria cases was significantly (p = 0.022) associated with the month of survey, highest during the rainy to dry season transition period (Fig. 3).

**Table 3:** Relationship between sociodemographic characteristics of study participants and malaria parasitaemia in the South West Region of Cameroon.

Variable	Subclass	% (n)	GMPD^#^	P value	Malaria parasitaemia prevalence				
					% (n)	Unadjusted P value	OR	95% CI	Adjusted P value
**Age groups (years)**	< 5	22.1 (177)	1822	**0.003**	24.9 (44)	**< 0.001**	3.55	2.08–6.05	**< 0.001**
	5–9	20.4 (163)	2324		37.4 (61)		5.80	3.40–9.90	**< 0.001**
	10–14	12.4 (99)	2134		25.3 (25)		2.76	1.34–5.68	**0.006**
	≥ 15	44.9 (359)	740^&^		8.6 (31)		Ref		
**Gender**	Male	43.8 (350)	2044	0.124	23.4 (82)	**0.040**	1.08	072–1.61	0.718
	Female	56.3 (450)	1440		17.6 (79)		Ref		
**Ethnicity**	Semi—Bantu	67.1 (537)	1771	0.713	19.0 (102)	0.595	0.69	0.43–1.27	0.139
	Bantu	18.5 (148)	1587		20.9 (31)		Ref		
**Locality**	Rural	25.5 (204)	1961	0.431	25.5 (52)	**0.027**	1.63	1.07–2.49	**0.023**
	Semi—Urban	74.5 (596)	1618		18.3 (109)		Ref		
**Altitude**	Low	23.0 (184)	2840	**0.040**	21.2 (39)	0.680	0.79	0.50–1.22	0.285
	Intermediate	77.0 (616)	1467		19.8 (122)		Ref		

OR = Odds Ratio; CI = Confidence Interval; Ref = Reference group; Boldface numbers indicate significant p values; ^#^GMPD = geometric mean parasite per microliter blood, ^&^Significantly lower than the corresponding values for participants < 5 (p = 0.022), 5–9 (p<0.001) and 10–14 (p = 0.014)

### ITN usage and malaria parasitaemia

The temporal distribution of Bednet use and *P*. *falciparum* parasitaemia prevalence is shown on Fig. 3. Overall, participants who did not sleep under ITN were more susceptible to malaria parasitaemia (adjusted OR = 1.70, 95%CI = 1.14–2.54, p = 0.009) after correction for age, locality, ethnicity. To avoid bias in this association due to a likely temporal variation in ITN usage and *P*. *falciparum* prevalence, the analysis was further stratified by season. The protective effect of ITN was still evident in the rainy season (adjusted OR = 1.93, 95%CI = 1.20–3.11, p = 0.006), after excluding the survey conducted in the rainy to dry season transition in Tole. There was, however, no association between ITN usage and malaria parasitaemia prevalence across the different age groups, gender, ethnicity and altitudes in both localities (Table 4). Nevertheless, malaria parasitaemia was lower in individuals of the Bantu ethnic group that used ITN compared to those who did not (p = 0.006) in semi-urban settings.

**Table 4:** Effect of ITN usage on malaria parasitaemia [% (n)] of study participants with different sociodemographic characteristics in the South West Region of Cameroon.

Variable	Semi-urban					Rural			
	Subclass	ITN use			P value	ITN use			P value
		n	No	Yes		n	No	Yes	
**Age groups (years)**	< 5	127	27.1 (13)	15.2 (12)	0.102	50	39.3 (11)	36.4 (8)	0.833
	5–9	118	39.0 (16)	33.8 (26)	0.570	45	46.2 (12)	36.8 (7)	0.532
	10–14	71	43.8 (7)	20.0 (11)	0.059	28	14.3 (2)	35.7 (5)	0.192
	≥ 15	280	10.4 (13)	7.1 (11)	0.131	79	11.4 (4)	6.8 (3)	0.372
**Gender**	Male	262	27.4 (26)	19.2 (32)	0.124	88	24.4 (11)	30.2 (13)	0.542
	Female	334	17.0 (23)	14.1 (28)	0.459	115	31.0 (18)	17.5 (10)	0.092
**Ethnicity**	Semi—Bantu	360	18.0 (29)	13.6 (27)	0.247	177	28.2 (24)	23.9 (22)	0.512
	Bantu	122	29.4 (20)	9.3 (5)	**0.006**	26	27.8 (5)	12.5 (1)	0.378
**Altitude**	Low	109	21.9 (16)	22.2 (8)	0.971	74	19.0 (4)	20.8 (11)	0.869
	Intermediate	487	21.0 (33)	15.8 (52)	0.153	129	30.5 (25)	25.5 (12)	0.549

## Discussion

ITNs have been shown to reduce morbidity and mortality, but coverage continues to be moderate in many parts of sub-Saharan Africa. Therefore, the malaria control community is shifting away from a narrow strategy of targeted ITN use amongst vulnerable groups such as children under 5 years old and pregnant women and towards universal coverage. The gains made through a nationwide free distribution system as well as the effect of ITNs on malaria prevalence were assessed in communities in south western Cameroon.

ITN ownership (69.3%) and usage by all persons (58.3%) has increased in the area after the campaign as reported elsewhere [22,24]. The free distribution campaign has therefore recorded moderate gains in the area as obtained elsewhere in the country [24] and Ethiopia [18,38,39]. Nevertheless, the percentage of children using ITNs is still below the World Health Assembly [27] and Ministry of Health [40] target of 80% for an acceptable level of protection. Low rates of bednet usage reported by communities in the tropics are attributed primarily to lack of sufficient nets to cover all household members [13,24] but also to heat discomfort associated with poor airflow caused by bed nets [41]. Universal coverage mass distribution campaigns will be needed to achieve maximal public health impact and a successful ‘keep up’ ITN distribution strategy needs to be supplemented with periodic ‘catch up’ campaigns. The government should, therefore, consider redistributing ITNs to increase the coverage and also conduct enhanced health education and community mobilization efforts to increase the possession and proper utilization of insecticide-treated bed nets.

ITN ownership and usage was lower in rural areas compared to semi-urban settings despite the free distribution through health facilities. This finding is in agreement with other studies [42–44] where inequalities were observed in ITNs ownership, with the poorest being disadvantaged. This pattern suggests that there might be barriers to persons from poorer communities obtaining ITNs. These barriers might include population differences such as socioeconomic status [45], reduced access to health facilities due to either distance or cost, poor quality health services and less functional ITN distribution system, or reduced knowledge about the health facility-based ITN distribution program [29]. On the other hand, the greater density of health facilities for resource distribution in urban areas [5,6] may account for the lower ITN possession in rural areas. An understanding of these dynamics is critical to evaluate current distribution programs as well as design future distribution strategies. These types of analyses as well as reports on both ITN possession and usage by all household members are critical in monitoring progress towards universal coverage and identifying gaps in coverage, such as low use by particular age groups.

In the south western region, the prevalence of malaria parasitaemia is still high (29.6%) in children of all age groups below 15 years, confirming that malaria remains a major cause of illness during childhood. Although higher malaria prevalence have been reported elsewhere [45], the age-specificity in prevalence from 24.9% for less than 5 year olds to 37.4% for children 5–9 years is in line with previous surveys in Mozambique where 39% of children 7–10 years had malaria parasitaemia [46]. Consequently, health education and treatment should not only target vulnerable groups (children under 5 and pregnant women), but all the age groups. The malaria parasitaemia prevalence was heterogeneous between localities ranging from 14.1 to 28.7%. Heterogeneity between and within villages has previously been reported [47] and is particularly a feature of areas that have undergone control activities [48]. Variations in malaria prevalence have also been reported in Bioko Island, Equatorial Guinea and North West Tanzania [22,49] after malaria control interventions. The transmission heterogeneity in this area suggests it may be beneficial to target hotspots for more frequent or concerted malaria control [50].

The greater risk of malaria parasite infection in rural settings reported here may be due to their specific characteristics that may facilitate human–mosquito contacts. This may accrue to the differences in housing types since house construction has previously been linked to malaria risk [33,45,51]. The predominance of plank houses in rural areas that have crevices for mosquitoe entry [52] and provide microenvironments conducive for mosquitoes, extends their chance of human contact and survival [51] unlike cement walls. House modification and improvement interventions are likely to be costly but should be considered as part of long term preventive measures. The keeping of farm animals, particularly pigs [45] and the absence of health facilities may also account for the higher malaria parasitaemia in rural settings. Malaria control programs should, therefore, pay particular attention to rural settings and consider advising owners of farm animals to ensure a reasonable separation between animal sheds and sleeping areas for humans.

ITNs have been shown to reduce morbidity and mortality in numerous controlled trials [8]. In our analysis, use of ITNs by participants was associated with reduced asexual parasitemia prevalence, a measure of malaria endemicity. This therefore, demonstrates a significant disease-specific impact of ITN use in the community. The use of ITNs as a community-level intervention, compared to untreated bednets or no bednet at all, not only directly prevents the mosquito from biting an individual, but kills the mosquito as well [30]. This reduces the mosquito infestation at household and community levels since more protection is gained from one or more ITNs in each household [4]. Given a total population of 20, 000,000 in the ten regions of Cameroon, based on the 2008 census, current levels of ITN use (58.3%) will be associated with an estimated 4,025,000 fewer individuals with asymptomatic parasitemia. The increased access to ITNs by the government of Cameroon through the free distribution campaign is a major step towards achieving the World Health Assembly and the RBM partnership goal of reducing the numbers of malaria cases and deaths recorded in 2000 by 75% or more by the end of 2015 [13]. There will, of course, be greater gains if ITN use is encouraged as a malaria control measure and universal coverage achieved with all children and adults sleeping under ITN.

This study has a number of limitations. First, it is a cross-sectional study, and inherently there are limitations. Though the sample size is large, Cameroon is a populous country and there is a definite possibility of sampling error. There are also possibilities of non-sampling errors depending on the interviewer, the questions and the respondents. As with all survey data, the findings are limited by recall and social desirability biases. Some answers to questions such as sleeping under ITNs were reported by the parents and not observed. Likewise, it was not possible for the team to verify the status of nets during the survey. However, the recall period for most questions was relatively short (e.g. the previous night), so that the response will reflect the prevailing circumstances. Secondly, the surveys were undertaken along the slope of Mount Cameroon in the South West Region, one of ten regions in Cameroon. Although, these localities are geographically diverse, the findings are not necessarily representative of the whole region or the country. Thirdly, malaria microscopy is subject to limitations in sensitivity at low parasitaemia typical of asymptomatic infections [53] which could have led to an underestimation of infection prevalence.

## Conclusion

Despite the free distribution campaigns, ITN ownership and usage, though improved, is still low. As children who reside in rural settings have greater disease burden (parasitemia) than children in semi-urban settings, the potential gains on both reducing inequities in ITN possession as well as disease burden might be substantial if equitable distribution strategies are adopted. Prior equity analyses of ITN distribution campaigns may therefore be necessary to reduce inequities in ITN possession. There may be more negative consequences on health of rural populations beyond the scope of this study due to disparities in access between rural and urban populations.
